# Arterial stiffness and its associations with left ventricular diastolic function according to heart failure types

**DOI:** 10.1186/s40885-022-00233-2

**Published:** 2023-03-15

**Authors:** Hack-Lyoung Kim, Jaehoon Chung, Seokmoon Han, Hyun Sung Joh, Woo-Hyun Lim, Jae-Bin Seo, Sang-Hyun Kim, Joo-Hee Zo, Myung-A Kim

**Affiliations:** 1 Division of Cardiology, Department of Internal Medicine, Seoul Metropolitan Government Seoul National University Boramae Medical Center, Seoul National University College of Medicine, Seoul, Republic of Korea; 2 Division of Cardiology, Department of Internal Medicine, National Medical Center, Seoul, Republic of Korea; 3 Department of Internal Medicine, Seoul Metropolitan Government Seoul National University Boramae Medical Center, Seoul, Republic of Korea

**Keywords:** Vascular stiffness, Diastolic function, Heart failure, Pulse wave analysis

## Abstract

**Background:**

Little is known about the characteristics of arterial stiffness in heart failure (HF). This study was performed to compare the degree of arterial stiffness and its association with left ventricular (LV) diastolic function among three groups: control subjects, patients with HF with reduced ejection fraction (HFrEF), and patients with HF with preserved ejection fraction (HFpEF).

**Methods:**

A total of 83 patients with HFrEF, 68 patients with HFpEF, and 84 control subjects were analyzed. All HF patients had a history of hospitalization for HF treatment. Brachial-ankle pulse wave velocity (baPWV) measurement and transthoracic echocardiography were performed at the same day in a stable condition.

**Results:**

The baPWV was significantly higher in patients with both HFrEF and HFpEF compared to control subjects (1,661 ± 390, 1,909 ± 466, and 1,477 ± 296 cm/sec, respectively; *P* < 0.05 for each). After adjustment of age, baPWV values were similar between patients with HFrEF and HFpEF (*P* = 0.948). In the multiple linear regression analysis, baPWV was significantly associated with both septal e′ velocity (β = –0.360, *P* = 0.001) and E/e′ (β = 0.344, *P* = 0.001). However, baPWV was not associated with either of the diastolic indices in HFrEF group. The baPWV was associated only with septal e′ velocity (β = –0.429, *P* = 0.002) but not with E/e′ in the HFpEF group in the same multivariable analysis.

**Conclusions:**

Although arterial stiffness was increased, its association with LV diastolic function was attenuated in HF patients compared to control subjects. The degree of arterial stiffening was similar between HFrEF and HFpEF.

**Supplementary Information:**

The online version contains supplementary material available at 10.1186/s40885-022-00233-2.

## Introduction

Heart failure (HF) is a terminal state of almost all heart diseases, and its prevalence continues to increase with age. Mortality and medical costs due to HF are so enormous that they pose a huge burden to our human society [1, 2]. Therefore, it is important to understand the underlying pathophysiology, and to develop a treatment that can prevent the occurrence of HF based on this. For several decades, many effective drugs for HF have been developed, which have greatly improved the survival rate of patients with HF [3–9]. However, since the mortality rate of HF is still very high, similar to that of some cancers, further efforts to treat HF are continuously required [1, 2].

HF is divided into HF with reduced ejection fraction (HFrEF) and HF with preserved ejection fraction (HFpEF) according to left ventricular ejection fraction (LVEF) [10]. Although HFpEF is also a clinically serious disease due to its high prevalence and poor prognosis, as in HFrEF, underlying pathophysiology and effective long-term treatment has not been well elucidated [11]. Recently, emerging evidence has shown that increased arterial stiffness plays an important role in the development of HFpEF [12–14]. However, most of the previous studies that conducted research into this issue analyzed subjects in the stage before clinically overt HF. Additionally, the role of arterial stiffness in HFrEF is still unknown. This study was performed to investigate in the degree of arterial stiffness and its association with left ventricular (LV) diastolic function in patients with HFrEF and HFpEF. We also compared results in patients with HF to control subjects without HF.

## Methods

### Study patients

This study is a cross-sectional study conducted at a general hospital in a large city (Seoul, Republic of Korea). The study was conducted in accordance with the Declaration of Helsinki. The study protocol was reviewed and approved by the Institutional Review Board of Seoul Metropolitan Government Seoul National University Boramae Medical Center (No. 10–2020-313). Written informed consent was obtained for prospectively enrolled subjects and informed consent was waived by Institutional Review Board for retrospectively enrolled subjects.

**Fig. 1:**
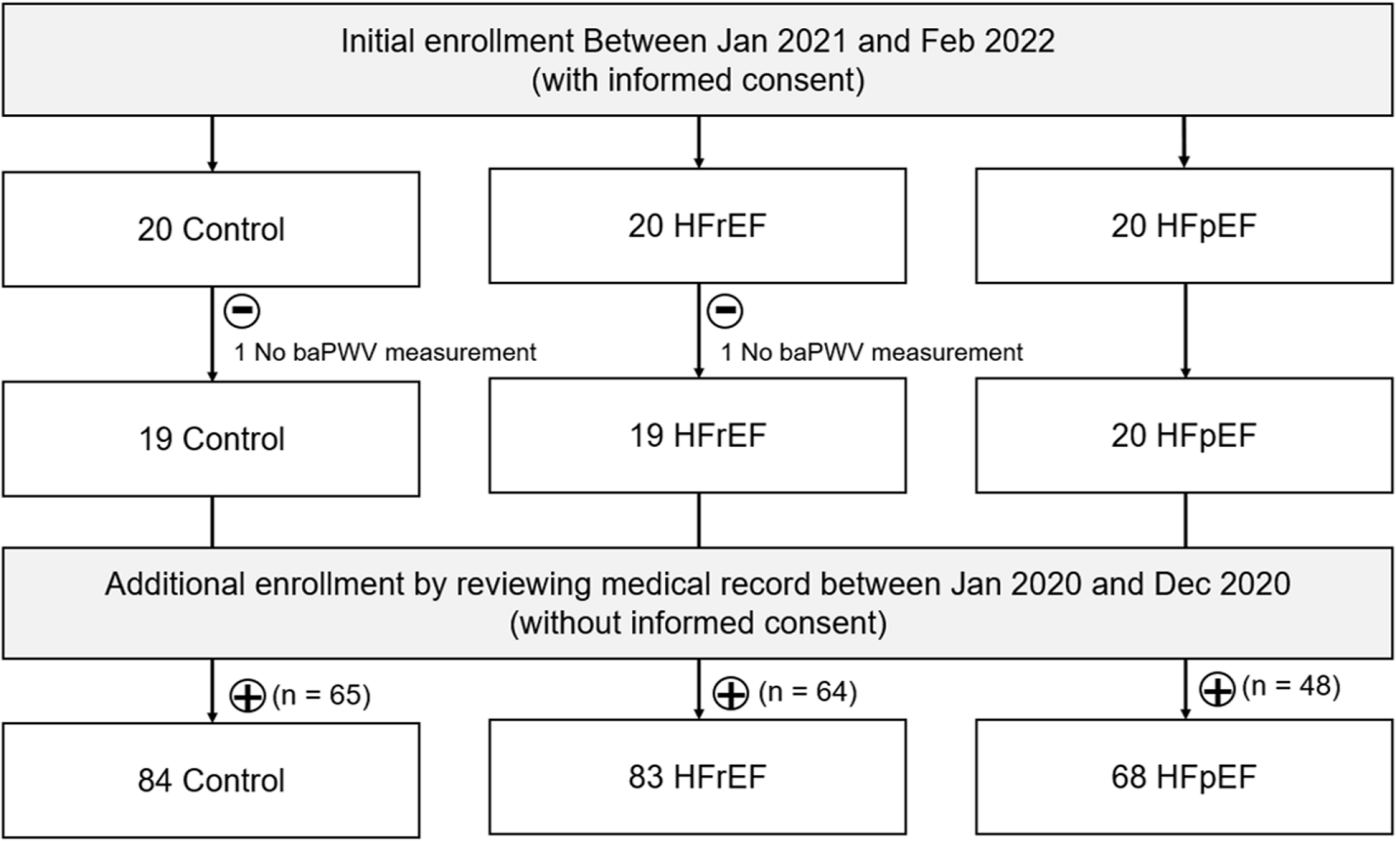
Flow chart for study subject enrollment. HFrEF, heart failure with reduced ejection fraction; HFpEF, heart failure with preserved ejection fraction; baPWV, brachial-ankle pulse wave velocity

In the HF groups, the eligible study subjects were patients who had a history of hospitalization for the management of new-onset acute HF or acute exacerbation of chronic HF. At the time of hospitalization, the main diagnosis should be HF. HF patients with LVEF < 40% were further stratified as the HFrEF group, and HF patients with LVEF ≥ 50% were further stratified as the HFpEF group [15]. Relatively healthy subjects without HF and other documented cardiovascular disease were enrolled as the control group. At the time of study enrollment, more than 30 days passed since HF hospitalization, and all study subjects were outpatient in a chronic stage with a stable condition. Both transthoracic echocardiography and brachial-ankle pulse wave velocity (baPWV) measurement were performed on the same day. Subjects with the following conditions were excluded: (1) uncontrolled HF symptoms with New York Heart Association dyspnea scale IV; (2) uncontrolled blood pressure with systolic blood pressure ≥ 180 mmHg, diastolic blood pressure ≥ 110 mmHg, or systolic blood pressure < 90 mmHg; (3) uncontrolled arrhythmia; (4) significant valvular dysfunction, moderate degree or more; (5) presence of pericardial effusion, maximal thickness > 10 mm, and (6) ankle-brachial index < 0.9. Initially 60 subjects (20 control, 20 HFrEF, and 20 HFpEF) were enrolled with informed consent between January 2021 and February 2022. Among them, one in the control group and one in the HFrEF group were excluded from the analysis because baPWV measurement was not performed. Additional 177 subjects (65 control, 64 HFrEF, and 48 HFpEF) were enrolled in the study through a retrospective review of their medical records between January and December 2020. Informed consent was not obtained from these subjects as data were collected retrospectively. Finally, 235 subjects (84 control, 83 HFrEF, and 68 HFpEF) were analyzed in this study. Flow chart for study enrollment is demonstrated in Fig. 1.

### Data collection

Body mass index was calculated as weight in kilograms divided by the square of height in meters (kg/m^2^). Systolic and diastolic blood pressures were recorded at the time of study enrollment using an oscillometric device. Information on cardiovascular risk factors including hypertension, diabetes mellitus, dyslipidemia, cigarette smoking status, coronary artery disease (CAD), and stroke were obtained. Hypertension was defined basis on previous diagnosis, the current use of antihypertensive medications used to control blood pressure, or blood pressure ≥ 140/90 mmHg. Diabetes mellitus was defined based on previous diagnosis, the current use of antidiabetic medications used to control hyperglycemia, or fasting blood glucose ≥ 126 mg/dL. Dyslipidemia was defined based on previous diagnosis, the current use of antidyslipidemic medications used to control dyslipidemia, or low-density lipoprotein cholesterol ≥ 160 mg/dL. Smokers were defined as those who had smoked during the past year. CAD included myocardial infarction and coronary revascularization. Stroke was defined as a sudden neurological abnormality with cerebral infarction or hemorrhage in imaging studies. After overnight fasting, venous blood was drawn and the blood levels of the following laboratory parameters were obtained: white blood cell count, hemoglobin, total cholesterol, low-density lipoprotein cholesterol, high-density lipoprotein cholesterol, triglyceride, creatinine, glucose, glycated hemoglobin, and C-reactive protein. Estimated glomerular filtration rate was calculated by the Modification of Diet in Renal Disease study equation. Information on concomitant cardiovascular medications including calcium channel blockers, beta blockers, renin-angiotensin system blockers, statins, and diuretics was also obtained.

### Transthoracic echocardiography

Transthoracic echocardiography was performed using commercially available machines (Vivid E9 and E95, GE Healthcare, Horten, Norway; EPIQ 7 and EPIQ CVx, Philips Ultrasound Inc., Bothell, WA, USA). Echocardiography was performed according to standardized protocols based on current guidelines’ recommendations [16, 17]. LV dimension was measured using M-mode echocardiography. LV ejection fraction was calculated using Simpson biplane method. LV mass (g) was calculated using the following formula: 0.8 × [1.04 × {(LV end-diastolic dimension) + (interventricular septal wall thickness) + (posterior wall thickness)}^3^ – (LV end-diastolic dimension)^3^] + 0.6. LV mass index was calculated as LV mass / body surface area. In apical four-chamber view, peak velocities of E and A waves of mitral inflow during diastole were obtained using a pulsed wave Doppler, and E/A ratio was calculated. Deceleration time of E wave was also measured. Using the tissue Doppler imaging technique, the peak velocity of mitral septal annulus (e′) was obtained. Left atrial (LA) volume was measured using the biplane disk summation method and indexed to body surface as LA volume index. In modified four-chamber view, the maximal velocity of tricuspid regurgitation (TR Vmax) was obtained using the continuous Doppler method. In this study, we focused on sepal e′ velocity and E/e′ as indicators of LV diastolic function because these indicators are relatively easy to measure and reliable indicators that are recommended first for the evaluation of left ventricular diastolic function [17]. Interobserver agreements of septal e′ and E/e′ were evaluated by Pearson correlation among 50 subjects. Correlation coefficients were 0.96 and 0.92 for e′ and E/e′, respectively, in our laboratory [18].

### Brachial-ankle pulse wave velocity measurement

Arterial stiffness was assessed using baPWV. The baPWV was measured noninvasively using a volume-plethysmography device (VP‐1000; Colin Co., Komaki, Japan) in the supine position 5 to 10 min after resting in an independent space in a quiet state [19, 20]. On the day of the test, cigarette smoking or consumption of beverages containing caffeine was restricted, and medications that were regularly taken were allowed. Arterial pulse wave was measured on both the brachial artery and posterior tibial artery of the subjects. During measurements, pulse volume waveform, blood pressure, and heart rate were recorded simultaneously. The baPWV were calculated as distance between the brachial and posterior tibial arteries divided by time interval. The distance between the brachial and posterior tibial arteries was estimated based on the height of the subject. The baPWV was measured on the left and right sides, and the average value was used in this study. All baPWV measurements were performed by a single trained operator. Coefficient of variation for intraobserver variability was 5.1% in our laboratory [21].

### Statistical analysis

Continuous variables were expressed as mean ± standard deviation and categorical variables were expressed as number (%). Comparisons among three groups (control, HFrEF, and HFpEF) were performed using analysis of variance (ANOVA) for continuous variables and chi-square test for categorical variables. Bonferroni post-hoc analysis was applied to compare the baPWV mean difference between the two groups. The difference in baPWV among the three groups was further compared by correcting for age through the analysis of covariance (ANCOVA). Linear relations between baPWV and diastolic parameters were assessed using Pearson correlation analysis. Scatter plots demonstrated these correlations. To find independent association between echocardiographic diastolic indices and baPWV, multiple linear regression analysis was performed. The following potential confounders were controlled during multivariable analysis: age, sex, and cardiovascular risk factors including hypertension, diabetes mellitus, and dyslipidemia, and smoking status. All analyses were two-tailed, and clinical significance was defined as *P* < 0.05. All statistical analyses were performed with the statistical package IBM SPSS ver. 23.0 (IBM Corp., Armonk, NY, USA).

## Results

Comparisons of clinical characteristics among three groups are shown in Table 1. In overall study patients, mean age was 67.0 ± 12.9 years, and 94 (40.0%) were female. The patients in the HFpEF group were oldest, and the proportion of female patients was highest. The HFpEF group had the highest systolic blood pressure as well as the highest prevalence of cardiovascular risk factors including hypertension, diabetes mellitus, dyslipidemia, previous history of CAD, and stroke. In laboratory findings, patients with HFpEF showed better cholesterol profiles and worse renal function compared to those with HFrEF and control groups. The blood levels of glucose, glycated hemoglobin and C-reactive protein were higher in both HFrEF and HFpEF group compared to control group. HF patients were taking more cardiovascular drugs than the control group. Beta blockers, renin-angiotensin system blockers, and diuretics showed the highest frequency of use in the HFrEF group and calcium channel blockers in the HFpEF group, but there was no difference in the statin use rate among the three groups.

**Table 1:** Clinical characteristics of study subjects

Characteristic	Control (*n* = 84)	HFrEF (*n* = 83)	HFpEF (*n* = 68)	*P*-value
Age (yr)	61.3 ± 11.1	64.6 ± 12.7	77.0 ± 9.2	< 0.001
Female sex	37 (44.0)	19 (22.9)	38 (55.9)	< 0.001
Body mass index (kg/m^2^)	24.5 ± 3.2	22.4 ± 7.3	24.3 ± 4.9	0.033
Systolic blood pressure (mmHg)	126.0 ± 13.0	124.0 ± 18.0	139.0 ± 22.0	< 0.001
Diastolic blood pressure (mmHg)	74.3 ± 9.4	76.2 ± 13.4	76.5 ± 11.6	0.436
Cardiovascular risk factor				
Hypertension	34 (40.5)	54 (65.1)	55 (80.9)	< 0.001
Diabetes mellitus	14 (16.7)	30 (36.1)	28 (41.2)	0.002
Dyslipidemia	30 (35.7)	25 (30.1)	33 (48.5)	0.062
Cigarette smoking	10 (11.9)	26 (31.3)	3 (4.4)	< 0.001
Coronary artery disease	0	13 (15.7)	13 (19.1)	0.001
Stroke	0	10 (12.0)	10 (14.7)	0.020
Laboratory finding				
White blood cell count (/µL)	6,136 ± 1,696	7,057 ± 1,865	6,494 ± 2,463	0.252
Hemoglobin (g/dL)	14.1 ± 1.3	15.9 ± 8.7	12.0 ± 2.0	< 0.001
Total cholesterol (mg/dL)	181.0 ± 41.0	166.0 ± 43.0	146.0 ± 62.0	< 0.001
LDL cholesterol (mg/dL)	108.0 ± 40.0	103.0 ± 41.0	87.0 ± 32.0	0.019
HDL cholesterol (mg/dL)	56.2 ± 13.1	39.7 ± 10.2	45.7 ± 13.5	< 0.001
Triglyceride (mg/dL)	114.0 ± 48.0	122.0 ± 68.0	128.0 ± 79.0	0.452
eGFR (mL/min/1.73m^2^)	94.6 ± 19.4	70.8 ± 26.5	65.9 ± 32.0	< 0.001
Glucose (mg/dL)	115.0 ± 31.0	123.0 ± 38.0	118.0 ± 34.0	0.659
Glycated hemoglobin (%)	5.91 ± 0.77	6.60 ± 1.20	6.38 ± 1.25	< 0.001
C-reactive protein (mg/dL)	0.21 ± 0.82	1.65 ± 4.31	1.09 ± 3.15	0.018
Cardiovascular medication				
Calcium channel blocker	9 (10.7)	22 (26.5)	33 (48.5)	< 0.001
Beta blocker	11 (13.1)	68 (81.9)	42 (61.8)	< 0.001
RAS blocker	10 (11.9)	70 (84.3)	37 (54.4)	< 0.001
Statin	31 (36.9)	27 (32.5)	33 (48.5)	0.122
Diuretics	6 (7.1)	46 (55.4)	31 (45.5)	< 0.001

Values are presented as number (%) or mean ± standard deviation

*HFrEF* Heart failure with reduced ejection fraction, *HFpEF* Heart failure with preserved ejection fraction, *LDL* Low-density lipoprotein, *HDL* High-density lipoprotein, *eGFR* Estimated glomerular filtration rate, *RAS* Renin-angiotensin system

Results of transthoracic echocardiography are demonstrated in Table 2. Patients with HFrEF had the largest LV systolic and diastolic dimensions and LV mass index. The mean LVEF were 67.4% ± 4.6%, 29.7% ± 6.4%, and 63.6% ± 8.1%, in the control, HFrEF, and HFpEF groups, respectively. Compared to the control group, LV diastolic function was more severely impaired in both patients with HFrEF and HFpEF, which was shown by lower septal e′ velocity as well as by higher E/e′, TR Vmax, and LA volume index.

**Table 2:** Echocardiographic findings of study subjects

Characteristic	Control (*n* = 84)	HFrEF (*n* = 83)	HFpEF (*n* = 68)	*P*-value
LV end-diastolic dimension (mm)	47.4 ± 3.6	55.8 ± 7.4	49.5 ± 5.1	< 0.001
LV end-systolic dimension, mm)	29.6 ± 3.1	43.8 ± 8.8	32.7 ± 5.7	< 0.001
LV ejection fraction (%)	67.4 ± 4.6	29.7 ± 6.4	63.6 ± 8.1	< 0.001
LV mass index (g/m^2^)	85.2 ± 17.8	144.0 ± 40.0	108.0 ± 30.0	< 0.001
E/A	0.80 ± 0.18	0.94 ± 0.55	0.89 ± 0.48	0.026
Deceleration time (ms)	223.0 ± 50.0	172.0 ± 55.0	184.0 ± 56.0	< 0.001
Peak septal e′ velocity (cm/sec)	6.27 ± 1.84	4.79 ± 1.75	5.56 ± 2.28	< 0.001
Septal E/e′	10.5 ± 3.4	16.4 ± 7.4	16.6 ± 7.2	< 0.001
Left atrial volume index (mL/m^2^)	31.8 ± 9.3	43.4 ± 18.2	57.3 ± 23.8	< 0.001
TR Vmax (m/sec)	2.28 ± 0.23	2.49 ± 0.55	2.66 ± 0.46	< 0.001

Values are presented as mean ± standard deviation

*HFrEF* Heart failure with reduced ejection fraction, *HFpEF* Heart failure with preserved ejection fraction, *LV* Left ventricular, *TR Vmax* Maximal velocity of tricuspid regurgitation

**Fig. 2:**
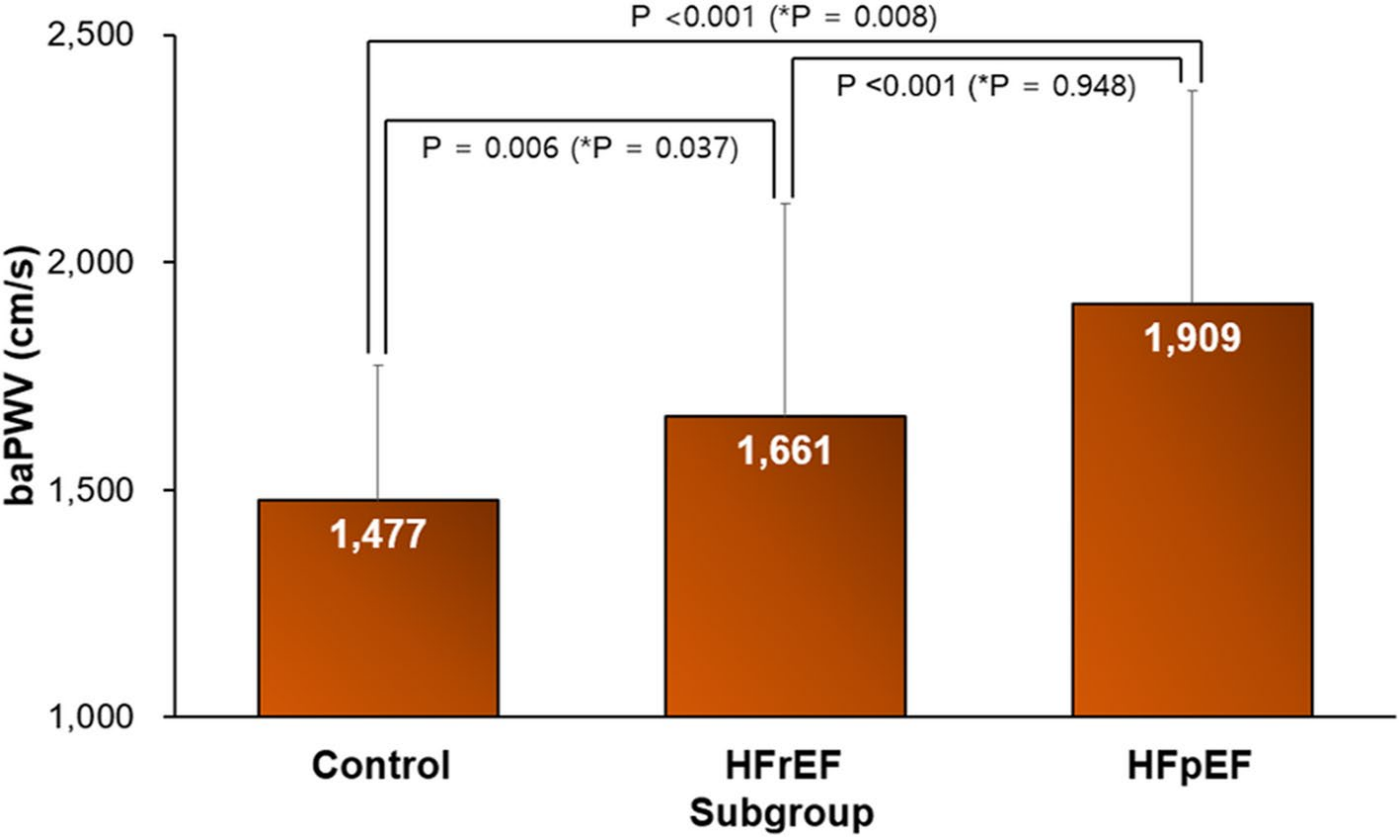
Brachial-ankle pulse wave velocity (baPWV) values according to heart failure types. HFrEF, heart failure with reduced ejection fraction; HFpEF, heart failure with preserved ejection fraction. ^*^Age-adjusted value through the analysis of covariance

Comparison of baPWV values among three groups is demonstrated in Fig. 2. Mean baPWV values were 1,477 ± 296, 1,661 ± 390, and 1,909 ± 466 cm/sec, in the control, HFrEF, and HFpEF groups, respectively (ANOVA, *P* < 0.001). In post-hoc analysis, baPWV value was significantly higher in patients with HFrEF than in control subjects (*P* = 0.006). Also, baPWV value was significantly higher in the HFpEF group compared to the control and HFrEF groups (*P* < 0.05 for each). Differences in baPWV values between the control group and HFrEF or HFpEF groups were also significant when age-adjusted using ANCOVA analysis (age-adjusted, *P* < 0.005). However, after adjusting for age, no difference in baPWV values was observed between the HFrEF and HFpEF groups (age-adjusted, *P* = 0.948). Without stratification by heart failure type, baPWV was significantly higher in heart failure group than in control group (1,771 ± 439 vs. 1,477 ± 297 cm/sec) even after controlling for age (age-adjusted, *P* < 0.001) (Supplementary Figure S1).

**Fig. 3:**
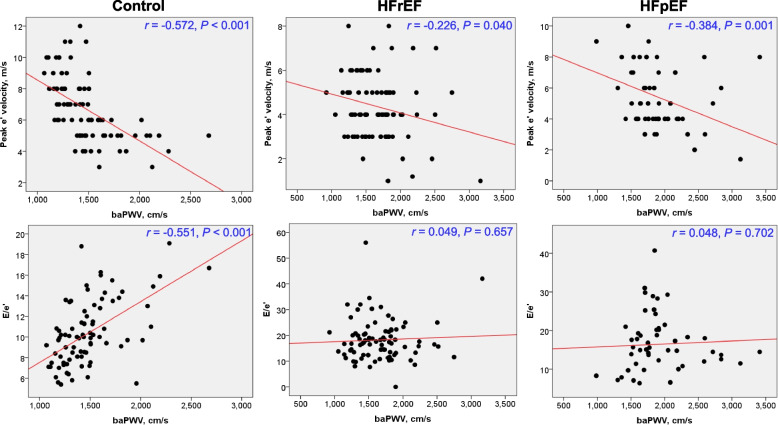
Linear correlations between brachial-ankle pulse wave velocity (baPWV) and echocardiographic diastolic indices according to heart failure types. HFrEF, heart failure with reduced ejection fraction; HFpEF, heart failure with preserved ejection fraction

Simple linear correlations between baPWV and diastolic parameters are shown in Fig. 3. The baPWV was significantly correlated with septal e′ velocity in all three groups (*r* = –0.572, *P* < 0.05 for the control group; *r* = –0.226, *P* = 0.040 for HFrEF group; *r* = –0.384, *P* = 0.001 for HFpEF group). baPWV was significantly correlated with E/e′ in control group (*r* = 0.551, *P* < 0.001), but not in the HFrEF (*r* = 0.049, *P* = 0.657) and HFpEF groups (*r* = 0.048, *P* = 0.702). Without stratification by heart failure type, baPWV was significantly correlated with septal e′ velocity (*r* = –0.213, P = 0.009) but not with septal E/e′ (*r* = –0.016, *P* = 0.842) (Supplementary Figure S2).

In the multiple linear regression analysis (Table 3), baPWV was significantly associated with both septal e′ velocity (β = –0.360, *P* = 0.001) and E/e′ (β = 0.344, *P* = 0.001) even after controlling for clinical confounders in the control group. However, baPWV was not associated with septal e′ velocity (β = –0.167, *P* = 0.177) and E/e′ (β = 0.063; *P* = 0.631) after controlling for confounders in the HFrEF group. The baPWV was associated with septal e′ velocity (β = –0.429, *P* = 0.002) but not with E/e′ (β = 0.117, *P* = 0.435) in the HFpEF group in the same multivariable analysis. Without stratification by heart failure type, baPWV was independently associated with septal e′ velocity (β = –0.281, *P* = 0.004) but not with E/e′ (β = 0.058, *P* = 0.551) (Supplementary Table S1).

**Table 3:** Independent association between brachial-ankle pulse wave velocity and left ventricular diastolic parameters

Dependent variable	β	*P*-value
Control group		
Septal e′ velocity	–0.360	0.001
E/e′	0.344	0.001
HFrEF group		
Septal e′ velocity	–0.167	0.177
E/e′	0.063	0.631
HFpEF group		
Septal e′ velocity	–0.429	0.002
E/e′	0.117	0.435

β and *P* values are for brachial-ankle pulse wave velocity. Following clinical covariates were controlled as potential confounders: age, sex, and cardiovascular risk factors including hypertension, diabetes mellitus, dyslipidemia, and cigarette smoking

*HFrEF* Heart failure with reduced ejection fraction, *HFpEF* Heart failure with preserved ejection fraction

## Discussion

Main findings of this study are as follows: (1) baPWV was significantly higher in patients with HFrEF and HFpEF compared to control subjects; (2) although univariable comparison showed that baPWV was significantly higher in patients with HFpEF than in those with HFrEF, it was similar between patients with HFrEF and HFpEF after adjusting for age; and (3) baPWV was significantly associated with septal e′ velocity and E/e′ in the control group, had no association with either of the LV diastolic indices in patients with HFrEF, and was associated only with septal e′ velocity, but not with E/e′ in patients with HFpEF.

Our results showed that baPWV was significantly higher in patients with HF than in control subjects who had no HF or other documented cardiovascular disease and stroke. The significance of this difference remains even after adjusting for age, a major determinant of arterial stiffness. It may be due to the fact that patients with HF had more various risk factors that could increase arterial stiffness compared to the control group, which was consistent with the characteristics of our study population. We also presented, for the first time, differences in the degree of arterial stiffness according to HF types. Although the baPWV value in the univariable comparison was higher in the HFpEF group than in the HFrEF group, there was no difference between the two groups after age adjustment. Large-scale data are required to verify our findings.

As arterial stiffness increases, LV diastolic function deteriorates. In a stiffened artery, the velocity of reflected wave is increased and merges with the forward wave early [22]. This raises systolic blood pressure and lowers diastolic blood pressure. Increased systolic blood pressure causes LV hypertrophy and decreased diastolic blood pressure reduces coronary perfusion. In addition, with the concept of a shared common risk factors, many cardiovascular risk factors related to arterial stiffening also exacerbate LV diastolic dysfunction [23]. Based on this hypothesis, many existing clinical studies have shown a significant association between increased arterial stiffness and LV diastolic dysfunction in the general population as well as in patients with certain diseases [18, 24–31]. However, in studies demonstrating such ventricular-arterial (VA) coupling, the study subjects were mostly restricted to the general population or subjects without documented cardiovascular disease including HF [25–27, 30]. To the best of our knowledge, there was only two studies showing the association between arterial stiffness and LV diastolic function in patients with established HF [28, 31]. Noguchi et al. [28] investigated 44 hypertensive patients with normal LVEF and 31 patients with reduced EF, and showed that cardio-ankle vascular index was correlated with septal e′ velocity in both groups. However, in that study, the definition of HFrEF and HFpEF depended only on LVEF and did not take into account clinical aspects such as hospitalization or symptoms. Additionally, the authors did not perform multivariable analysis [28]. More recently, another study of 107 patients with HFpEF revealed that ambulatory arterial stiffness index was correlated with E/e′ [31]. In our study, baPWV was correlated with e′ velocity in patients with HFpEF but not in those with HFrEF. It seems that the results of each individual study are inconsistent due to differences in the basic characteristics of the study subjects, including race and the method of measuring arterial stiffness. Our study is most meaningful in that it showed a relationship between arterial stiffness and LV diastolic function according to HF types and compared it with the control group.

In the comparisons between control and HF groups, our results showed that the degree of arterial stiffness is more severe in the HF groups, but the association between arterial stiffness and LV diastolic function was stronger in the control group. This implies that arterial stiffness has a greater impact on the diastolic function of LV in the stage before the HF onset, and that the effect is somewhat weakened when HF has already occurred. Therefore, this may suggest that strategy targeting arterial stiffness to improve LV diastolic function or prevent HF [32] should be implanted as early as possible. In addition, baPWV was associated with septal e′ velocity in patients with HFpEF but not in patients with HFrEF. This may be a consistent finding with the results of previous studies showing the important role of arterial stiffness in the development of HFpEF [32]. Our study also showed that baPWV was not associated with E/e′ in either type of HF. It has been suggested that e′ velocity is less affected by the LV loading condition than E/e′; thus, e′ velocity is a more reliable indicator of LV diastolic function [33]. Septal e′ may be a better indicator for response monitoring than E/e′ in treatment strategies targeting arterial stiffness especially in patients with HFpEF.

Our study has several limitations. First, the associations of baPWV with septal e′ velocity and E/e′ were determined with cross-sectional data; therefore, the causal relationship between arterial stiffness and diastolic function could not be confirmed. Second, although carotid-femoral pulse wave velocity (cfPWV) is the gold standard for the non-invasive measurement of arterial stiffness [34], baPWV was used in our study. However, baPWV is more simple to measure and has a good correlation with cfPWV [23]. Third, the unavoidable differences in clinical characteristics among the three groups might affect the study results. In order to overcome this, we enrolled consecutive subjects who visited the same institution during the same period and corrected for important confounding variables through multivariable analysis. Fourth, due to the small number of heart failure patients enrolled in the study, it was difficult to conduct a detailed analysis according to the etiology of heart failure, such as ischemic vs. non-ischemic. Finally, because our study target is limited to Korean adults, it may be difficult to directly apply our findings to other ethnic groups.

## Conclusions

Compared to the control subjects, arterial stiffness was increased in HF patients, but the association of the arterial stiffness on LV diastolic function was weaker in HF patients compared to the control subjects. These results suggest early detection and effective intervention for reverse arterial stiffening may limit adverse cardiac remodeling and HF. The degree of arterial stiffness was similar between HFrEF and HFpEF, but the association between arterial stiffness and LV diastolic function was stronger in the HFpEF group. Given that baPWV correlated well with septal e′ velocity in HFpEF, septal e′ velocity could be useful for devising a therapeutic strategy targeting VA coupling. Further large-scale studies are needed to confirm our findings.

## Supplementary Information

**Additional file 1:** **Supplementary Figure S1.** The difference in baPWV between control and HF group. baPWV was significantly higher in HF patients than in control subjects. **Supplementary Figure S2.** Associations between baPWV and LV diastolic parameters in control and HF group. The associations of baPWV with septal e′ velocity and septal E/e′ were stronger in control subjects than in HF patients. **Supplementary Table S1.** Independent association between brachial-ankle pulse wave velocity and left ventricular diastolic parameters.

## Data Availability

The datasets used and/or analyzed during the current study are available from the corresponding author on reasonable request.

## Abbreviations

ANCOVA: Analysis of covariance
ANOVA: Analysis of variance
baPWV: Brachial-ankle pulse wave velocity
CAD: Coronary artery disease
cfPWV: Carotid-femoral pulse wave velocity
eGFR: Estimated glomerular filtration rate
HDL: High-density lipoprotein
HF: Heart failure
HFpEF: Heart failure with preserved ejection fraction
HFrEF: Heart failure with reduced ejection fraction
LA: Left atrial
LDL: Low-density lipoprotein
LV: Left ventricular
LVEF: Left ventricular ejection fraction
RAS: Renin-angiotensin system
TR Vmax: Maximal velocity of tricuspid regurgitation
VA: Ventricular-arterial
