# Supplementary material for: Arterial stiffness and its associations with left ventricular diastolic function according to heart failure types
Source: Clin Hypertens. 2023 Mar 15;29:8. doi: 10.1186/s40885-022-00233-2 (PMC10015827; doi:10.1186/s40885-022-00233-2)
Supplement: Supplementary file 1 — Additional file 1: Supplementary Figure S1. The difference in baPWV between control and HF group. baPWV was significantly higher in HF patients than in control subjects. Supplementary Figure S2. Associations between baPWV and LV diastolic parameters in control and HF group. The associations of baPWV with septal e′ velocity and septal E/e′ were stronger in control subjects than in HF patients. Supplementary Table S1. Independent association between brachial-ankle pulse wave velocity and left ventricular diastolic parameters. [file 40885_2022_233_MOESM1_ESM.docx]

**Supplementary Data**

**Supplementary Figure S1.** The difference in baPWV between control and HF group. baPWV was significantly higher in HF patients than in control subjects.


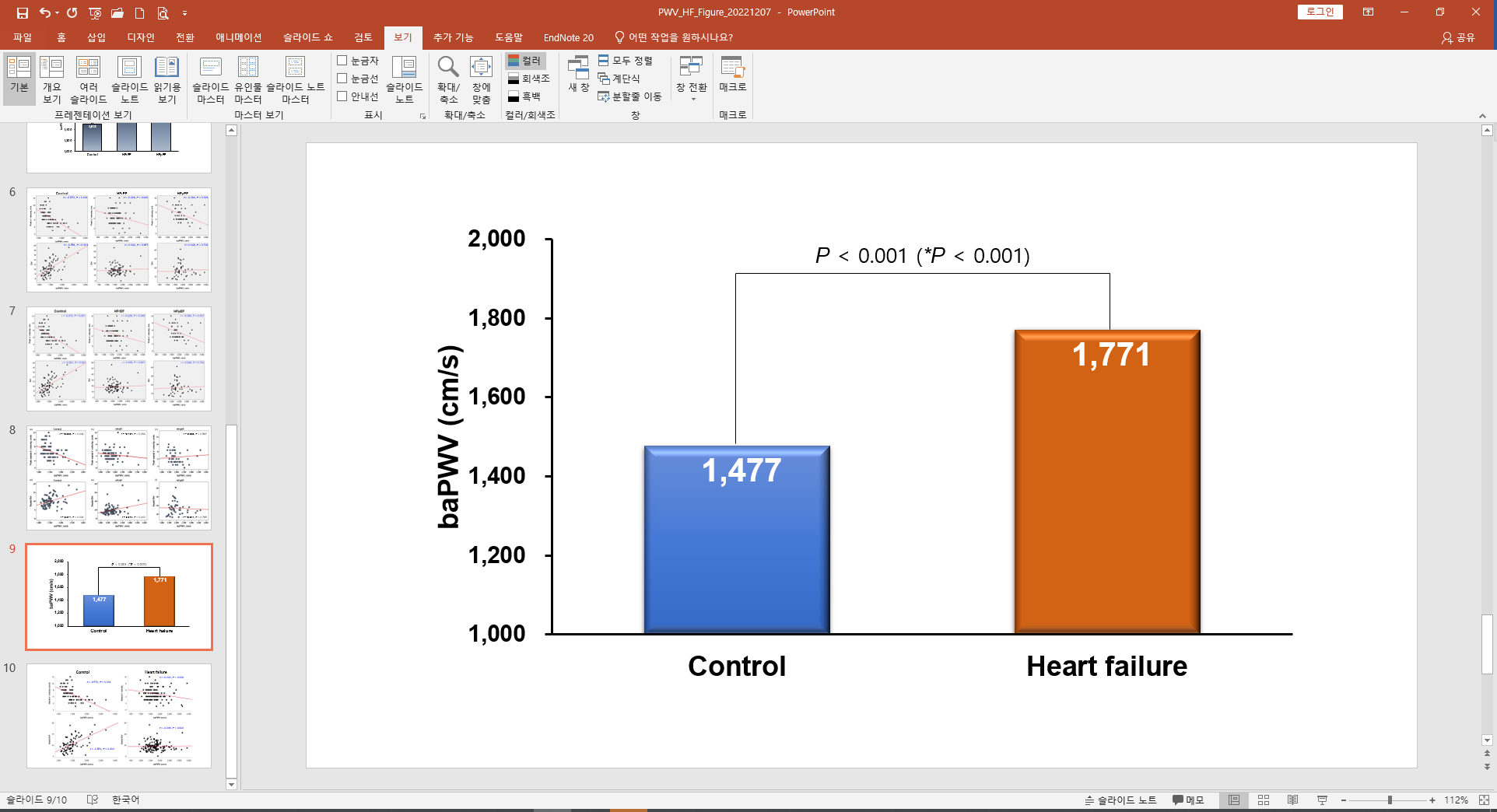


Supplementary Figure S2. Associations between baPWV and LV diastolic parameters in control and HF group. The associations of baPWV with septal e′ velocity and septal E/e′ were stronger in control subjects than in HF patients.


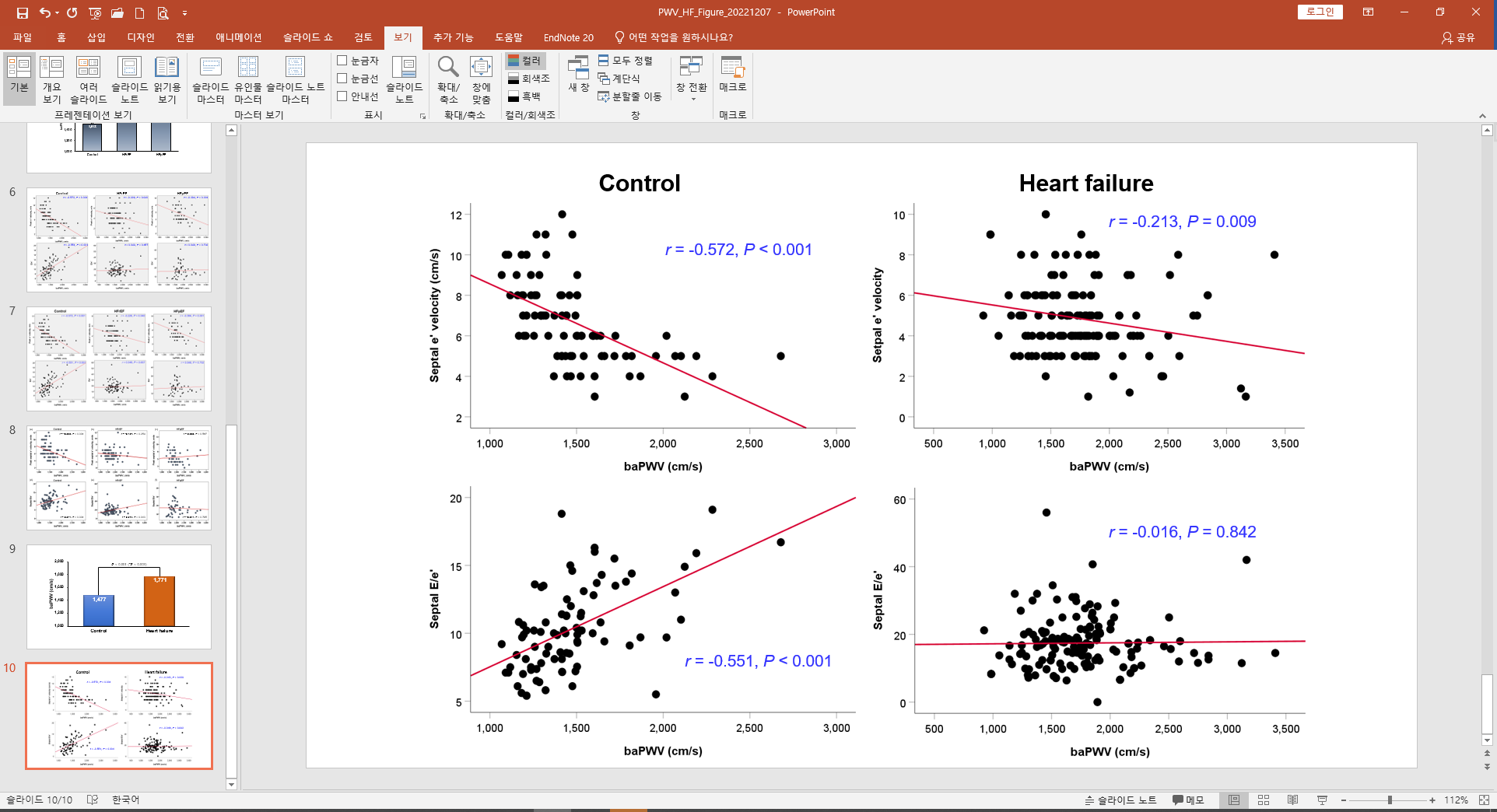


**Supplementary Table S1. Independent association between brachial-ankle pulse wave velocity and left ventricular diastolic parameters**

| **Dependent variable** | ***β*** | ***P* value** |
| --- | --- | --- |
| **Control group** |  |  |
| Septal e′ velocity | -0.360 | 0.001 |
| Septal E/e′ | 0.344 | 0.001 |
| **Heart failure group** |  |  |
| Septal e′ velocity | -0.281 | 0.004 |
| Septal E/e′ | 0.058 | 0.551 |

*β* and *P* values are for brachial-ankle pulse wave velocity. Following clinical covariates were controlled as potential confounders: age, sex and cardiovascular risk factors including hypertension, diabetes mellitus, dyslipidemia and cigarette smoking.
